# Effect of the Children’s Healthy Living Program on Young Child Overweight, Obesity, and Acanthosis Nigricans in the US-Affiliated Pacific Region: A Randomized Clinical Trial

**DOI:** 10.1001/jamanetworkopen.2018.3896

**Published:** 2018-10-26

**Authors:** Rachel Novotny, James Davis, Jean Butel, Carol J. Boushey, Marie Kainoa Fialkowski, Claudio R. Nigg, Kathryn L. Braun, Rachael T. Leon Guerrero, Patricia Coleman, Andrea Bersamin, Aufai Apulu Ropeti Areta, Leroy R. Barber, Tayna Belyeu-Camacho, Joshua Greenberg, Travis Fleming, Elise Dela Cruz-Talbert, Ashley Yamanaka, Lynne R. Wilkens

**Affiliations:** 1 Department of Human Nutrition, Food and Animal Science, College of Tropical Agriculture and Human Resources, University of Hawaii at Manoa, Honolulu; 2 Office of Biostatistics and Quantitative Health Sciences, John A. Burns School of Medicine, University of Hawaii at Manoa, Honolulu; 3 Nutrition Support Shared Resource, University of Hawaii Cancer Center, Honolulu; 4 Office of Public Health Studies, Myron B. Thompson School of Social Work, University of Hawaii at Manoa, Honolulu; 5 College of Natural and Applied Sciences, University of Guam, Mangilao; 6 Cooperative Research and Extension Education Services, Northern Marianas College, Saipan, Northern Mariana Islands; 7 Department of Biology and Wildlife, University of Alaska, Fairbanks; 8 Agriculture, Community and Natural Resources Division, American Samoa Community College, Pago Pago; 9 Division of Agriculture and Life Sciences, College of Natural and Applied Sciences, University of Guam, Mangilao; 10 School of Natural Resources and Extension, University of Alaska, Fairbanks; 11 Department of Social Sciences, University of Hawaii at West Oahu, Kapolei; 12 Biostatistics and Informatics Shared Resource, University of Hawaii Cancer Center, Honolulu

## Abstract

**Importance:**

Pacific Islanders have among the highest rates of obesity and type 2 diabetes in the world. Targeting children is critical for primary prevention.

**Objectives:**

To prevent young child overweight and obesity and to improve health in the US-Affiliated Pacific region via the Children’s Healthy Living Program.

**Design, Setting, and Participants:**

In this multijurisdictional, multilevel, multicomponent community randomized clinical trial, where all evaluable children were analyzed according to the random assignment of their community, hierarchical difference-in-difference models accounted for the community randomization, community clustering with jurisdictions, and these models were adjusted for the age and sex distribution of the community. The setting was 27 communities in 5 jurisdictions (Alaska, American Samoa, Commonwealth of the Northern Mariana Islands, Guam, and Hawaii). Participants were 4329 children (time 1) and 4042 children (time 2) aged 2 to 8 years in 27 selected communities from October 7, 2012, to October 25, 2015. Data analysis was completed in June 2018.

**Interventions:**

Nineteen activities addressed policy, environment, messaging, training, and 6 target behaviors (sleep time, screen time, physical activity, fruits and vegetables, water, and sugar-sweetened beverages).

**Main Outcomes and Measures:**

Primary outcomes were body size measurements. Secondary outcomes were acanthosis nigricans, sleep quality and duration, dietary intake, physical activity, and other questionnaire reponses.

**Results:**

The study included 27 communities and 8371 evaluable children (mean [SD] age, 5.4 [1.8] years; 50.9% male [n = 4264]). Data analysis included 952 children in the intervention group and 930 children in the control group aged 2 to 5 years at time 1; 825 children in the intervention group and 735 children in the control group aged 2 to 5 years at time 2; 565 children in the intervention group and 561 children in the control group aged 6 to 8 years at time 1; and 517 children in the intervention group and 560 children in the control group aged 6 to 8 years at time 2. The intervention communities showed significant improvement compared with control communities in overweight and obesity prevalence (effect size [*d*] = −3.95%; 95% CI, −7.47% to −0.43%), waist circumference (*d* = −0.71 cm; 95% CI, −1.37 to −0.05 cm), and acanthosis nigricans prevalence (*d* = −2.28%; 95% CI, −2.77% to −1.57%). Age and sex subgroup analysis revealed greater difference among the intervention communities in acanthosis nigricans prevalence in the group aged 2 to 5 years (−3.99%) vs the group aged 6 to 8 years (−3.40%), and the interaction was significant (*d* = 0.59%, *P* < .001), as well as the smaller difference in the group aged 2 to 5 years (−0.10%) vs the group aged 6 to 8 years (−1.07%) in screen time (*d* = −0.97 hour per day, *P* = .01).

**Conclusions and Relevance:**

The intervention reduced the prevalence of young child overweight and obesity and acanthosis nigricans. Comprehensive, effective, and sustainable interventions are needed to improve child health in the US-Affiliated Pacific region.

**Trial Registration:**

ClinicalTrials.gov Identifier: NCT01881373

## Key Points

**Question:**

Does a multijurisdictional, multilevel, multicomponent community intervention decrease young child overweight and obesity in the US-Affiliated Pacific region?

**Findings:**

Among 27 communities and 8371 children in this randomized clinical trial, the Children’s Healthy Living Program decreased overweight and obesity prevalence by 3.95% among children aged 2 to 8 years in the US-Affiliated Pacific region.

**Meaning:**

A multilevel, multicomponent approach reduced young child overweight and obesity.

## Teaser

This randomized clinical trial evaluates the effect of an intervention that aims to prevent young child overweight and obesity and to improve health in the US-Affiliated Pacific region via the Children’s Healthy Living Program.

## Introduction

The rates of obesity and type 2 diabetes among adults in the Pacific are among the highest in the world.^1^ Prevention is needed, and starting in childhood is the best time for prevention because childhood obesity and type 2 diabetes track into adulthood.^2^ Obesity among young children in the US-Affiliated Pacific (USAP) region jurisdictions was 14% in 2013 and was higher among children aged 6 to 8 years than among those aged 2 to 5 years.^3^ Young child obesity was found to be highly associated with occurrence of acanthosis nigricans, an indicator of insulin resistance,^4,5^ pointing to the clinical significance of obesity, even in childhood.

Behavior change interventions alone have not been sufficient to reduce the prevalence of obesity or type 2 diabetes. Broad changes in the obesogenic environment are needed to influence population health, including obesity.^6^ A comprehensive literature review^7^ found 18 effective interventions for children aged 2 to 10 years, intervening on home, school, and/or community levels. Bright Start,^8^ Shape Up Somerville,^9^ and Romp & Chomp^10^ interventions targeted eating and physical activity through multilevel interventions addressing various aspects of the school environment and/or policy and measured influence on child overweight or obesity.^8,9,10^ Bright Start intervention showed a statistically significant 10% decrease in obesity prevalence.^8^ Shape Up Somerville intervention showed a statistically significant decrease in the body mass index (BMI) *z* score among children aged 3 years by 0.10 U.^9^ Romp & Chomp intervention showed lower mean weight, BMI, and BMI *z* score in the subsample aged 3.5 years and lower prevalence of overweight and obesity in the subsamples aged 2 years and 3.5 years than in the control group.^10^ Not included in the comprehensive literature review^7^ and most similar to the Children’s Healthy Living Program (CHL) in design is the newer Identification and Prevention of Dietary- and Lifestyle-Induced Health Effects in Children and Infants (IDEFICS) multilevel study^11^ conducted in multiple communities in 8 European countries. The IDEFICS intervention did not achieve significant change in the BMI *z* score, waist-to-height ratio, or body fat percentage.^12^ No overall significant behavioral changes were seen either.^11^ Therefore, multilevel environmental community interventions show promising but mixed results in reducing overweight and obesity in young children, although, to our knowledge, there are no multilevel studies to date among Native Hawaiian, Pacific Islander, and Alaskan Native populations. For replication, dissemination, and sustainability, methodological work is needed to understand what did and did not work in these complex interventions.

The CHL intervention was built using a social ecological framework of health and wellness. It was designed to act on multiple levels and multiple components of the behavioral, physical, social and cultural, and economic and policy environment^13^ to influence prevalence of child overweight and obesity and risk for acanthosis nigricans in multiple USAP region communities.

Guided by the Analysis Grid for Elements Linked to Obesity (ANGELO) framework,^14^ the intervention team designed the multilevel intervention by merging community input and evidence-based strategies identified in the literature.^7,13,15^ The CHL sought to identify and build on what was currently working in communities by engaging community partners and members in ways sensitive to their culture and to put the health and well-being of young children at the forefront of community decisions and actions in a way that could be sustained through the land grant college framework and local coalitions.^16^ The CHL trial used a unique template for implementation and had exceptional accuracy of anthropometric measurements. The CHL trial was also novel in the diversity and range of unstudied races/ethnicities and places in a single trial, including populations who are at high risk for chronic disease yet have a dual burden of undernutrition and overnutrition. The trial protocol is available in Supplement 1. Our hypothesis was that the CHL multijurisdictional, multilevel, multicomponent community randomized clinical trial would reduce young child overweight and obesity in the USAP region (eAppendix in Supplement 2).

## Methods

### Study Population

In 2011, a group of 27 communities in 5 USAP jurisdictions (Alaska, American Samoa, Commonwealth of the Northern Mariana Islands, Guam, and Hawaii) were selected using the following criteria based on the 2000 US Census: more than 15% of the population was of indigenous/native descent (native to each jurisdiction), more than 10% of the population was younger than 10 years, the population size exceeded 1000, and accessibility was reasonable (Table 1). Eighteen selected communities were matched for size and randomized within jurisdiction strata to intervention or to control (a delayed optimized intervention arm) according to the Consolidated Standards of Reporting Trials (CONSORT) reporting guideline (Figure). Another 9 communities were selected for temporal assessment (only BMI, waist circumference, and selected demographics were assessed). A total of 4333 children aged 2 to 8 years were recruited for evaluation measurements at schools or community events in selected jurisdictions at time 1 (baseline), with 4048 children recruited at time 2 (24 months) (Figure). Sample size was determined before the intervention based on anthropometry, accelerometry, and food records. A recruitment goal of 180 children per community was established based on these estimates, as published by Wilkens et al.^17^ The samples at time 1 and time 2 were independent samples of children from the communities (not repeated measures). The interval between measurements averaged 26.0 months (range across communities, 21.2-28.6 months). From October 7, 2012, to October 25, 2015, a total of 4329 children in the target age group of 2 to 8 years were included in this analysis from time 1 (Table 2) and 4042 children from time 2. Table 2 lists the sample characteristics by intervention period. eTable 1 in Supplement 2 lists the sample sizes by measurement module collected.

**Table 1.** Children’s Healthy Living Program Community Characteristics by Study Participants at Time 1 (Baseline), US-Affiliated Pacific Region, 2012-2013

**Community Cluster**	**Study Group**	**2010 US Census Community Cluster Population**	**Indigenous Population, %**	**Median Annual Household Income, $**^a^	**Implemented Intervention Activities Counts**^b^
**Alaska**					
Fairbanks	Intervention	97 581	27	47 500	97
Mat-Su	Control	88 995	21	27 500	NA
Kenai	Temporal	49 733	44	47 500	NA
Anchorage	Temporal	291 826	58	27 500	NA
**American Samoa**					
Aua/Leloaloa/Atu’u	Intervention	2884	97	7500	85
Tula/Alao/Aoa	Control	1755	100	7500	NA
Fagaitua/Alofau/Masefau	Intervention	1504	100	7500	62
Aoloau/Malaeloa/Aasu	Control	2357	98	7500	NA
Tafuna	Temporal	7945	99	7500	NA
Pavaiai	Temporal	2450	87	11 250	NA
**Commonwealth of the Northern Mariana Islands**					
Kagman	Intervention	5 184	92	7500	130
Koblerville/San Antonio	Control	5593	35	7500	NA
Tanapag/San Roque	Intervention	3308	67	15 000	114
Oleai	Control	5472	45	7500	NA
Garapan	Temporal	9096	24	7500	NA
Tinian and Rota	Temporal	5663	68	7500	NA
**Guam**					
Yona/Talafofo	Intervention	6480	80	15 000	92
Agat/Santa Rita	Control	8720	74	27 500	NA
Yigo	Intervention	20 539	39	15 000	153
Sinajana/Agana Heights	Control	6310	79	15 000	NA
Dededo	Temporal	44 943	53	15 000	NA
**Hawaii**					
Nanakuli/Maili (Oahu)	Intervention	22 154	76	27 500	275
Waimanalo (Oahu)	Control	9932	79	15 000	NA
Hilo (Hawaii)	Intervention	43 263	82	47 500	92
Wailuku (Maui)	Control	20 729	55	47 500	NA
Kauai	Temporal	67 091	38	47 500	NA
Molokai	Temporal	4503	63	15 000	NA

Abbreviation: NA, not applicable.

^a^ Median of household income midpoint variable.

^b^ Counts include repeated activities.

**Figure.**
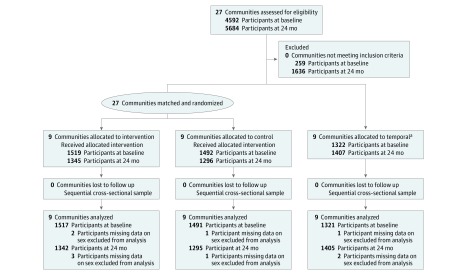
CONSORT 2010 Flow Diagram ^a^Temporal communities were selected in each jurisdiction to monitor changes in child obesity over time, without influence of the Children’s Healthy Living Program.

**Table 2.** Children’s Healthy Living Program Child Characteristics by Study Period and Group, US-Affiliated Pacific Region, 2012-2015

Characteristic	No. (%)						
	Intervention		Control		Temporal^a^		Total
	Time 1^b^	Time 2^c^	Time 1^b^	Time 2^c^	Time 1^b^	Time 2^c^	
**Total participants**	1517	1342	1491	1295	1321	1405	8371
**Age Group, y**							
2-5	952 (62.8)	825 (61.5)	930 (62.4)	735 (56.8)	996 (75.4)	930 (66.2)	5368 (64.1)
6-8	565 (37.2)	517 (38.5)	561 (37.6)	560 (43.2)	325 (24.6)	475 (33.8)	3003 (35.9)
**Sex**							
Boys	757 (49.9)	674 (50.2)	771 (51.7)	660 (51.0)	701 (53.1)	701 (49.9)	4264 (50.9)
Girls	760 (50.1)	668 (49.8)	720 (48.3)	635 (49.0)	620 (46.9)	704 (50.1)	4107 (49.1)
**Hispanic Ethnicity**							
Yes	173 (11.4)	92 (6.9)	136 (9.1)	123 (9.5)	120 (9.1)	129 (9.2)	773 (9.2)
No	1340 (88.3)	1250 (93.1)	1355 (90.9)	1172 (90.5)	1197 (90.6)	1276 (90.8)	7590 (90.7)
Unknown	4 (0.3)	0	0	0	4 (0.3)	0	8 (0.1)
**Race/Ethnicity**							
White	120 (7.9)	138 (10.3)	147 (9.9)	139 (10.7)	151 (11.4)	182 (13.0)	877 (10.5)
Black	6 (0.4)	17 (1.3)	1 (0.1)	7 (0.5)	8 (0.6)	9 (0.6)	48 (0.6)
Asian	85 (5.6)	74 (5.5)	195 (13.1)	159 (12.3)	197 (14.9)	204 (14.5)	914 (10.9)
Native Hawaiian, Pacific Islander	898 (59.2)	774 (57.7)	809 (54.3)	687 (53.1)	599 (45.3)	506 (36.0)	4273 (51.0)
American Indian, Alaskan Native	24 (1.6)	9 (0.7)	19 (1.3)	8 (0.6)	81 (6.1)	79 (5.6)	220 (2.6)
>1	374 (24.7)	318 (23.7)	317 (21.3)	283 (21.9)	282 (21.3)	409 (29.1)	1983 (23.7)
Unknown	10 (0.7)	12 (0.9)	3 (0.2)	12 (0.9)	3 (0.2)	16 (1.1)	56 (0.7)
**Indigenous**^**d**^							
Yes	1087 (71.7)	871 (64.9)	968 (64.9)	759 (58.6)	793 (60.0)	852 (60.6)	5330 (63.7)
No	430 (28.3)	471 (35.1)	523 (35.1)	536 (41.4)	528 (40.0)	553 (39.4)	3041 (36.3)

^a^ Temporal communities were selected in each jurisdiction to monitor changes in child obesity over time, without influence of the Children’s Healthy Living Program.

^b^ Participants enrolled at baseline.

^c^ Participants enrolled at 24 months.

^d^ Native race/ethnicity of jurisdiction.

Institutional review board approval was obtained from the University of Hawaii at Manoa (Honolulu), University of Guam (Mangilao), and University of Alaska (Fairbanks). Northern Marianas College (Saipan, Northern Mariana Islands) and American Samoa Community College (Pago Pago) ceded approval to the University of Hawaii at Manoa. Written informed consent and assent were obtained from the caregiver and assent from the child participant.

### CHL Intervention

The CHL intervention was developed by a consortium of collaborators at Pacific land grant universities.^18^ The CHL intervention package consisted of a common template of 19 activities, which were selected to address target behaviors derived from community-informed ideas^16^ and blended with approaches from successful interventions from the literature.^13^ Implementation focused on supporting existing community programs to expand or innovate (positive deviance approach). The intervention activities were grouped into the following 4 crosscutting functions (or strategies): organizational policy change, environmental change, social marketing, and training (Table 3). These strategies also addressed the interpersonal (training role models, parents, and teachers), community (increasing access to healthy foods and environments for safe play), and organizational and policy (strengthening preschool wellness policies) levels of the social ecological model. The intervention spanned a 2-year period, with monthly process measures collected and used to calculate dose and fidelity^19^ in each of the 9 intervention communities (Table 3).

**Table 3.** Children’s Healthy Living Program Intervention Template, Including Activities, Behavioral Outcomes, and Crosscutting Function Dose, US-Affiliated Pacific Region, 2012-2015

Activity	Behavioral Outcomes						Crosscutting Function Dose^a^
	Increase Sleep Time	Decrease Screen Time	Increase Physical Activity	Increase Fruits and Vegetables	Increase Drinking Water	Decrease Sugar-Sweetened Beverages	
**Policy: Review Assessment Data for Policy and Physical Environment Related to the 6 CHL Target Behaviors**							**4.95**
Review preschool wellness policy assessment data to identify training needs	X	X	X	X	X	X	NA
Review community assessment data to identify areas for advocacy			X	X	X	X	NA
**Environment: Community Partnership and Advocacy for Environmental Change**							**33.95**
Work with coalitions to advocate for:							NA
Better access to parks that are safe and inviting			X				NA
Better access to clean water					X	X	NA
Safer environments for walking and biking			X				NA
Better food placement in stores				X	X	X	NA
Gardens and hydroponics			X	X			NA
Partner with existing entities to purchase or obtain sponsorship for:							NA
Water in the preschools					X	X	NA
Gardening supplies for preschool kids			X	X			NA
Sports equipment for preschool kids			X				NA
Campaigns and messages	X	X	X	X	X	X	NA
**Messaging: Promote the CHL Message to the Community**							**23.59**
Support role models to deliver CHL messages in various venues	X	X	X	X	X	X	NA
Enhance existing social marketing campaigns related to the 6 CHL target behaviors	X	X	X	X	X	X	NA
Advertise CHL or other activities that promote the 6 CHL target behaviors	X	X	X	X	X	X	NA
**Capacity Building: Train the Trainers/Role Models**							**25.37**
Train individuals to promote gardening in preschools and communities	X	X	X	X	X	X	NA
Train individuals to lead interactive, hands-on, and family-based sessions	X	X	X	X	X	X	NA
Train care providers (preschool or home care) on wellness policies	X	X	X	X	X	X	NA
Train care providers (preschool or home care) in curricula related to the 6 CHL target behaviors	X	X	X	X	X	X	NA
Train role models (community champions, role celebrities, role models)	X	X	X	X	X	X	NA

Abbreviations: CHL, Children’s Healthy Living Program; NA, not applicable.

^a^ Crosscutting function dose calculation is the sum of each activity dose in the crosscutting function in the intervention communities. Activity dose is the number of activities conducted times the effectiveness score times the total number of participants divided by intended number of participants.

The CHL subsequently (after the time 2 measures were completed) implemented intervention activities that worked the best (using fidelity and qualitative data) with the control communities, which we referred to as a delayed optimized intervention (eTables 2 and 3 in Supplement 2). Using the Reach Effectiveness Adoption Implementation Maintenance (RE-AIM) framework,^20^ implementation fidelity assessment data, and local CHL qualitative data, the activities were ranked on the following criteria: (1) community acceptability of the activity, (2) reach the activity had on the target audience, (3) likelihood of effectiveness of the activity, (4) adoption of the activity by community partners, (5) sustainability of the activity in the community, and (6) feasibility of implementing the activity in a 6-month time frame. The resulting optimized community plan included 8 activities, with at least 1 activity from each CHL crosscutting function, and addressed the multiple social and environment levels.

### Measures, Training, and Standardization

Key measurement tools and outcome measures were derived from the literature,^21^ and staff were trained in protocols and standardized in anthropometric measurements. To ensure consistency of protocol across sites, a quality assurance protocol was implemented during both data collection periods.^22^ Data were collected on demographics, child body size, functional outcomes of obesity, and lifestyle behavior, which included sleep time, screen time, physical activity, and food intake. Measures included anthropometry, food and activity logs, and visual inspection of the neck for acanthosis nigricans. Surveys and inventories of food and physical activity resources in the community were also conducted and will be assessed in future analyses as mediators of the intervention. The study outcomes listed below were measured for children across jurisdictions using a shared method.

### Primary Outcomes of Body Size Measurements

Body size measurements included weight, height, and waist circumference and the resultant calculations of BMI, BMI *z* score, BMI percentile, and percentage overweight and obese relative to National Center for Health Statistics data.^23,24^ Trained staff in all jurisdictions used standard instruments, including scales for weight (seca 876), stadiometers for height (model PE-AIM-101; Perspective Enterprises), and tape measures for waist circumference (seca 201). Overweight was defined as the 85th to 94th percentiles for the BMI *z* score (with BMI calculated as weight in kilograms divided by height in meters squared), and obesity was defined as at least the 95th percentile for the BMI *z* score. Before measuring children for the study, all measurers had to display good agreement compared with an expert,^22^ as determined per guidelines by Zerfas^25^ during training sessions on anthropometry.

### Secondary Outcomes

#### Acanthosis Nigricans

Acanthosis nigricans as a skin indicator of insulin resistance^5^ was assessed at the back of the neck by trained staff using the scale developed by Burke et al.^26^ Acanthosis nigricans is highly associated with obesity and is a key health outcome that indicates the clinical importance of overweight and obesity. Type 2 diabetes is highly prevalent in the USAP region, such that a state of emergency for chronic disease was declared May 24, 2010.

#### Sleep Quality and Duration

Sleep quality was measured with the Tayside Children’s Sleep Questionnaire.^27^ Sleep duration was reported by the caregiver as hours asleep at night or during naps.

#### Dietary Intake

We calculated food groups of the children’s diet from 2 dietary records on randomly selected days to ensure representation of all days of the week across children. The records were completed by the parent or caregiver, with assistance from other child caregivers, as previously described.^28^ These data were entered into the PacTrac3 applications, a Pacific Tracker 2 update.^29^ We used the food composition database that was developed and is maintained by the Nutrition Support Shared Resource at the University of Hawaii Cancer Center to include information on local foods identified by our group from children in the Pacific region.^30^ The dietary components were averaged across days, weighted for weekday and weekend days, and adjusted for within-person variance.^31^

#### Physical Activity

We measured physical activity using the objective measure from omnidirectional accelerometers (Actical; Philips Respironics) and a questionnaire on screen time, regarded as sedentary behavior and a lifestyle measure.^32^ The accelerometers were selected based on good agreement between the devices and observation in a pilot test of the devices in young children in year 1 of the CHL.^33^ The CHL coordinating center at the University of Hawaii at Manoa trained staff at each jurisdiction on the use of the accelerometers before measurement began. Children were instructed to wear the accelerometers for 6 days without removal. The accelerometers were set to record children’s movements, known as counts, at each second. The sum of accelerometer counts over a 60-second interval was used to classify the minute into categories of sedentary (0-40 counts), light (41-2295 counts), moderate (2296-6815 counts), and vigorous (>6815 counts) physical activity based on cut points from the manufacturer. Sleep was included in the sedentary grouping. The number of minutes per category was summed for each day, overall, and within sustained bouts of 5 minutes and then averaged across days, weighted for weekdays and weekend days.

#### Other Questionnaires

Parent and caregiver respondents for the children completed questionnaires about other demographics. These included race/ethnicity, categorized according to the US Office of Management and Budget (OMB) guidelines and by our own indigenous variable, defined as the native racial/ethnic group of the jurisdiction where the data were being collected, alone or with other races/ethnicities.

### Statistical Analysis

Sleep durations less than 5 hours per day were excluded, as were biologically implausible BMI and waist circumference *z* scores greater than −8 SDs and less than 4 SD based on guidance from the National Health and Nutrition Examination Survey reference values.^23,24^ Overweight and obesity was compared with healthy weight (underweight was excluded). Accelerometry was found to display high reactivity in the first few days, so those results were dropped, and presented results are based on days 4 to 6. Accelerometry data are given in 5-minute bouts.

Hierarchical difference-in-difference models were used to estimate the means at each time point by randomization group. The models accounted for the study design using community as the unit for hypothesis testing and accounting for the community clusters with jurisdiction strata. The models were adjusted for age (in months) and sex and were weighted to account for the number of children in the community. Race/ethnicity was not adjusted for because it was highly collinear with jurisdiction. Models of dichotomous outcomes of overweight and obesity prevalence and acanthosis nigricans used a logistic link, while models of continuous outcomes of the BMI *z* score, waist circumference, and each target behavior (sleep time and sleep disturbance, screen time, physical activity, fruits and vegetables, water, and sugar-sweetened beverages) used a linear link. Assumptions of the linear statistical models were checked; if not met, Box-Cox transformations were applied for the outcome. If results were similar, untransformed models are displayed. Transformations were used in final models for waist circumference, sleep disturbance, screen time, and water intake. Changes by randomization group over time and the difference in changes between groups (the effect size [*d*]) were assessed by a Wald test, with *df* based on the number of communities. The models were run overall and by subgroups defined by age and sex; the performance of the intervention between subgroups was tested based on a Wald test of cross-product terms between randomization group and subgroup membership. Two-sided *P* < .05 was considered statistically significant.

## Results

### Demographics

Among 8371 participants CHL-wide (mean [SD] age, 5.4 [1.8] years; 50.9% male [n = 4264]), 64.1% (n = 5368) of children were in the group aged 2 to 5 years, while 35.9% (n = 3003) of children were in the group aged 6 to 8 years (Table 2), with 49.1% (n = 4107) being girls. Sixty-four percent (n = 5330) of children were classified as indigenous to their jurisdiction (Alaskan Natives [Yupik and Inupiaq] for Alaska, Samoan for American Samoa, Chamorro and Carolinian for Commonwealth of the Northern Mariana Islands, Chamorro for Guam, and Native Hawaiian for Hawaii). The percentage indigenous (63.7% [n = 5330]) is higher than the 51.0% (n = 4273) classified as Native Hawaiians and Pacific Islanders by the OMB classification because many of the indigenous individuals were classified as more than 1 race/ethnicity by the OMB, and the OMB does not specify if the Native Hawaiian and Pacific Islander group is the same as the jurisdiction’s ancestral group.

Data analysis included 952 children in the intervention group and 930 children in the control group aged 2 to 5 years at time 1; 825 children in the intervention group and 735 children in the control group aged 2 to 5 years at time 2; 565 children in the intervention group and 561 children in the control group aged 6 to 8 years at time 1; and 517 children in the intervention group and 560 children in the control group aged 6 to 8 years at time 2.

### Primary Outcomes

Obesity-related outcomes of the CHL intervention communities are listed in Table 4. The CHL trial yielded significant differences between the intervention and control communities over time of −3.95% (95% CI, −7.47% to −0.43%) for overweight and obesity prevalence (−3.90% [95% CI, −6.32% to −1.47%] vs 0.05% [95% CI, 0.00% to 0.11%]; *P* = .02) and −0.71 cm (95% CI, −1.37 to −0.05 cm) for waist circumference (−0.12 vs 0.59 cm; *P* = .02). The overall mean BMI *z* score changed by −0.06 SD U (95% CI, −0.13 to 0.01 SD U) in the intervention communities compared with −0.01 SD U (95% CI, −0.06 to 0.05 SD U) in the control communities, which was not significantly different (−0.06 SD U; *P* = .20).

**Table 4.** Children’s Healthy Living Program Trial Mean Changes in Primary and Secondary Outcomes, US-Affiliated Pacific Region, 2012-2015^a^

Child Measure	No.^b^	Intervention				Control				Intervention vs Control	
		Time 1, Baseline (95% CI)	Time 2, 24 mo (95% CI)	Difference (95% CI)	*P* Value	Time 1, Baseline (95% CI)	Time 2, 24 mo (95% CI)	Difference (95% CI)	*P* Value	Difference (95% CI)	*P* Value
**Primary Outcome Anthropometry Measurements**											
Overweight/obesity prevalence, %	7944	31.02 (27.03 to 35.31)	27.12 (23.65 to 30.89)	−3.90 (−6.32 to −1.47)	.02	29.90 (25.67 to 34.51)	29.95 (25.99 to 34.24)	0.05 (0.00 to 0.11)	.35	−3.95 (−7.47 to −0.43)	.02
BMI *z* score	7944	0.66 (0.54 to 0.77)	0.60 (0.49 to 0.70)	−0.06 (−0.13 to 0.01)	.09	0.61 (0.48 to 0.75)	0.61 (0.49 to 0.73)	−0.01 (−0.06 to 0.05)	.84	−0.06 (−0.14 to 0.03)	.20
Waist circumference, cm	7992	52.31 (51.58 to 53.07)	52.14 (50.89 to 53.48)	−0.12 (−0.72 to 0.49)	.65	51.75 (50.92 to 52.63)	52.34 (51.42 to 53.32)	0.59 (0.33 to 0.85)	<.001	−0.71 (−1.37 to −0.05)	.02
**Secondary Outcomes**											
Acanthosis nigricans prevalence, %	5585	3.54 (1.42 to 8.57)	0.96 (0.45 to 2.02)	−2.58 (−4.00 to −1.17)	<.001	2.19 (1.05 to 4.51)	1.89 (0.96 to 3.70)	−0.30 (−2.28 to 1.78)	.60	−2.28 (−2.77 to −1.57)	<.001
Sleep time, h/d^c^	7699	9.71 (9.43 to 9.99)	9.78 (9.44 to 10.12)	0.07 (−0.24 to 0.38)	.66	10.00 (9.80 to 10.20)	10.25 (9.79 to 10.72)	0.25 (−0.10 to 0.60)	.15	−0.19 (−0.72 to 0.35)	.48
Sleep disturbance score, range of 1-9^c^	5499	6.10 (5.61 to 6.62)	5.70 (5.08 to 6.36)	−0.41 (−0.96 to 0.14)	.07	6.44 (5.76 to 7.18)	6.82 (5.99 to 7.73)	0.38 (−0.59 to 1.36)	.22	−0.79 (−1.90 to 0.32)	.08
Screen time, h/d^c^	5494	3.71 (3.22 to 4.24)	3.56 (3.10 to 4.05)	−0.16 (−0.69 to 0.38)	.28	3.75 (3.21 to 4.35)	4.05 (3.41 to 4.75)	0.30 (−0.36 to 0.96)	.19	−0.45 (−1.18 to 0.28)	.11
Accelerometer total METs, min/d^d^	2951	29.22 (29.05 to 29.39)	29.02 (28.81 to 29.23)	−0.20 (−0.42 to 0.01)	.07	29.06 (28.80 to 29.32)	28.97 (28.79 to 29.15)	−0.09 (−0.20 to 0.03)	.13	−0.11 (−0.39 to 0.16)	.38
Moderate/vigorous physical activity, min/d	2951	19.11 (15.91 to 22.30)	19.94 (16.95 to 22.93)	0.83 (−2.40 to 4.06)	.59	17.56 (14.19 to 20.92)	17.23 (14.41 to 20.05)	−0.32 (−2.07 to 1.42)	.70	1.16 (−2.89 to 5.20)	.55
Sedentary/light physical activity, min/d^e^	2951	1346.33 (1336.45 to 1356.22)	1347.05 (1335.95 to 1358.16)	0.72 (−8.56 to 9.99)	.87	1350.13 (1338.21 to 1362.04)	1351.36 (1343.09 to 1359.62)	1.23 (−5.86 to 8.32)	.71	−0.51 (−14.49 to 13.47)	.94
Fruits, cups/d	4763	1.02 (0.90 to 1.14)	0.95 (0.85 to 1.05)	−0.08 (−0.16 to 0.07)	.07	1.08 (0.97 to 1.19)	1.04 (0.90 to 1.17)	−0.05 (−0.10 to 0.01)	.09	−0.03 (−0.13 to 0.07)	.55
Vegetables, cups/d	4763	0.59 (0.52 to 0.66)	0.58 (0.53 to 0.64)	−0.01 (−0.05 to 0.04)	.73	0.61 (0.54 to 0.69)	0.61 (0.56 to 0.66)	0.00 (−0.05 to 0.05)	.89	−0.004 (−0.08 to 0.07)	.90
Water, cups/d^c^	4763	1.22 (1.04 to 1.40)	1.28 (1.35 to 1.44)	0.07 (−0.20 to 0.34)	.30	1.27 (1.11 to 1.44)	1.38 (1.23 to 1.53)	0.10 (0.00 to 0.12)	.01	−0.03 (−0.16 to 0.09)	.71
Sugar-sweetened beverages, cups/d	4763	0.62 (0.49 to 0.75)	0.57 (0.45 to 0.69)	−0.05 (−0.11 to 0.01)	.09	0.62 (0.39 to 0.84)	0.56 (0.38 to 0.73)	−0.06 (−0.22 to 0.09)	.40	0.01 (−0.15 to 0.17)	.86

Abbreviation: BMI, body mass index; METs, metabolic equivalent tasks.

^a^ Means are based on a mixed model with a linear link for continuous outcomes and a logistic link for dichotomous outcomes that accounts for the randomization unit of community and the community clusters within jurisdiction strata, weights for the number of children in each community, and adjusts for child’s age and sex. *P* values are based on a Wald test, with *df* based on the number of communities.

^b^ Number of children included in analysis.

^c^ The variables were back transformed from the regression model.

^d^ Number of minutes per day within bouts of 5 minutes, averaged over 4 to 6 days of accelerometer use.

^e^ Sedentary/light includes sleep activity.

### Secondary Outcomes

There was a significant difference between the intervention and control communities over time of −2.28% (95% CI, −2.77% to −1.57%) for acanthosis nigricans prevalence (−2.58% vs −0.30%; *P* < .001). Age and sex subgroup analysis revealed a greater difference among the intervention communities in acanthosis nigricans prevalence in the group aged 2 to 5 years (−3.99%) vs the group aged 6 to 8 years (−3.40%), and the interaction was significantly different (*d* = 0.59%; *P* < .001) (eFigure in Supplement 2).

None of the behavioral variables showed significant overall differences between the intervention and control communities in similarly constructed models (Table 3), including sleep time (hours per day), physical activity (moderate/vigorous and sedentary/light hours per day), fruits and vegetables (cups per day), water (cups per day), and sugar-sweetened beverages (cups per day). Consumption of sugar-sweetened beverages declined over time by similar amounts in both the intervention and control communities. Screen time decreased in the intervention communities and increased in the control communities, but the overall differences were not significant. However, the difference in screen time of −1.07% (−0.45 vs 0.63 hour per day, *P* = .004) was significant in the intervention vs control communities among the older children (age range, 6-8 years) (eFigure in Supplement 2), and the interaction with age was significant (*P* = .01). There was also a smaller difference in the group aged 2 to 5 years (−0.10%) vs the group aged 6 to 8 years (−1.07%) in screen time (d = −0.97 hour per day, P = .01).

## Discussion

When the intervention communities were compared with the control communities, the CHL randomized clinical trial demonstrated significant improvement, with a 3.95% reduction in overweight and obesity prevalence, a 0.71-cm lower waist circumference, and a 2.28% decrease in acanthosis nigricans prevalence among children aged 2 to 8 years. The prevalence changes were the same or greater than the difference of 3% overweight and obesity prevalence achieved in Romp & Chomp^10^ among preschoolers in Australia but were less than those seen in Bright Start.^8^ The CHL resulted in a reduction in the BMI *z* score by 0.06 SD U, although the change was not significantly different between the intervention and control communities. The BMI *z* score effect size was similar to but smaller than the difference seen in Shape Up Somerville,^9^ which achieved a difference of 0.10 U in children aged 3 years.

While the behavioral targets of the CHL did not show significant differences when tested individually, the positive obesity-related outcomes are likely a result of nudges or shifts in multiple behavioral measures,^34^ including some that we did not evaluate, such as other foods, stress, or the gut microbiome, which likely changed with the CHL intervention as well.^35^ Furthermore, age and sex subgroups showed changes in sleep disturbance and screen time. We expect there was movement to light activity from sedentary behavior that was not well captured owing to reactivity to the accelerometers and inability of the accelerometers to distinguish sedentary and light activity. Because of logistics, cost, and participant burden, all behavioral components were also assessed with a smaller sample size than the primary outcomes; therefore, loss of power may also have had a role in those measures not showing significant change. There was an increase in water intake and a decrease in sugar-sweetened beverage consumption in both the intervention and control groups that did not reach significance but may have contributed to the effect. To further understand how the intervention achieved the results, we plan to further develop and examine measures of the dose of the intervention and its components in future analyses.

### Limitations and Strengths

Limitations of the CHL trial included bleed of the intervention activities into control communities (a delayed optimized intervention), as well as other health messaging campaigns that were jurisdiction-wide, which may have dampened the effect of the trial, although this ultimately supported positive public health change in the region. The community randomized clinical trial design reduced power. Using the number of communities as the unit for hypothesis testing likely is conservative^36^; other statistical methods will also be further examined in future analysis of the results. While we conducted a feasibility study of children wearing wrist accelerometers,^33^ the accelerometry data did not reveal expected differences in the CHL intervention, and further exploration of whether the tools are effectively capturing the movements made by young children is needed. While we implemented strong diet methodology, the sample size was necessarily smaller, and recording of child intake by caregivers has limitations.

Strengths of our intervention included high-quality standardized anthropometry measurement and a common intervention template derived from a blend of community and evidence-based approaches and actions, which provided flexibility for each community to localize and tailor the intervention to build on and strengthen local initiatives, circumstances, and indigenous culture. The intervention approach harnessed the motivation and sense of belonging to a Pacific region–wide collective with the empowerment of local communities, leveraging both local and regional tools for sustainability. Land grant institutions allowed a common system (backbone) and common understanding, provided assessment and evaluation resources, and supported reach into (extension) and sustainability in communities. The CHL intervention further harnessed and was strengthened by indigenous Pacific cultures that value belonging to and action for their indigenous group.^37^ Our intervention built on positive aspects of the community and supported the community to expand its initiatives, strengthening existing partnerships. This collaboration built on previous work. We used a program steering committee with a local leader to facilitate collaboration, along with frequent conference calls.

## Conclusions

The obesity-related community changes observed in the CHL trial support that multiple levels and multiple components of intervention are needed to change the trajectory of childhood obesity in communities. The CHL intervention also had a number of important unanticipated positive effects that are vital for sustainability and long-term change, which will be tracked and examined in further study and analysis. These positive effects included creation of coalitions and ongoing professional development among individuals involved in the CHL.

Regarding public health implications, multiple levels and multiple components of the intervention activities together changed the policy, environmental, and systems context in which child overweight and obesity and acanthosis nigricans occurred. The CHL regional multilevel, multicomponent community randomized clinical trial slowed gain in waist circumference and reduced the prevalence of overweight, obesity, and acanthosis nigricans among young children in the USAP region.

## Notes

Supplement 1. Trial Protocol

Supplement 2. **eAppendix.** Supplemental Appendix **eTable 1.** Sample Size by Measurement Modules and by Intervention Time Period **eTable 2.** CHL Optimized Intervention of 8 Activities and 6 Behavioral Targets **eTable 3.** Children’s Healthy Living Program Trial Mean Changes in Primary and Secondary Outcomes Between Intervention and Temporal Communities, US-Affiliated Pacific Region, 2012-2015 **eFigure.** Children’s Healthy Living (CHL) Program (a) Acanthosis Nigricans and (b) Screen Time Comparisons Between Intervention and Control Groups With Age and Sex Interaction *P* Values, US-Affiliated Pacific, 2012-2015

Supplement 3. Data Sharing Statement

## References

[1] Hawley NL, McGarvey ST Obesity and diabetes in Pacific Islanders: the current burden and the need for urgent action. Curr Diab Rep. 2015;15(5):. doi:10.1007/s11892-015-0594-5 25809458

[2] Cameron N, Demerath EW Critical periods in human growth and their relationship to diseases of aging. Am J Phys Anthropol. 2002;45(suppl 35):159-. doi:10.1002/ajpa.10183 12653312

[3] Novotny R, Fialkowski MK, Li F, Systematic review of prevalence of young child overweight and obesity in the United States–Affiliated Pacific region compared with the 48 contiguous states: the Children’s Healthy Living Program.Am J Public Health. 2015;105(1):e22-e35. doi:10.2105/AJPH.2014.302283 25393168 PMC4265894

[4] Novotny R, Li F, Fialkowski MK, ; Children’s Healthy Living (CHL) Program Prevalence of obesity and acanthosis nigricans among young children in the Children’s Healthy Living Program in the United States Affiliated Pacific. Medicine (Baltimore). 2016;95(37):e4711. doi:10.1097/MD.0000000000004711 27631218 PMC5402561

[5] Styne DM, Arslanian SA, Connor EL, Pediatric obesity: assessment, treatment, and prevention: an Endocrine Society clinical practice guideline. J Clin Endocrinol Metab. 2017;102(3):709-757. 28359099 10.1210/jc.2016-2573 PMC6283429

[6] Winett LB, Wulf AB, Wallack L Framing strategies to avoid mother-blame in communicating the origins of chronic disease. Am J Public Health. 2016;106(8):1369-1373. doi:10.2105/AJPH.2016.303239 27310351 PMC4940651

[7] Nigg CR, Ul Anwar MM, Braun K, A review of promising multicomponent environmental child obesity prevention intervention strategies by the Children’s Healthy Living Program. J Environ Health. 2016;79(3):18-26. 29120137

[8] Story M, Hannan PJ, Fulkerson JA, Bright Start: description and main outcomes from a group-randomized obesity prevention trial in American Indian children. Obesity (Silver Spring). 2012;20(11):2241-2249. doi:10.1038/oby.2012.89 22513491 PMC3407274

[9] Economos CD, Hyatt RR, Goldberg JP, A community intervention reduces BMI *z*-score in children: Shape Up Somerville first year results. Obesity (Silver Spring). 2007;15(5):1325-1336. doi:10.1038/oby.2007.155 17495210

[10] de Silva-Sanigorski AM, Bell AC, Kremer P, Reducing obesity in early childhood: results from Romp & Chomp, an Australian community-wide intervention program. Am J Clin Nutr. 2010;91(4):831-840. doi:10.3945/ajcn.2009.28826 20147472

[11] De Bourdeaudhuij I, Verbestel V, De Henauw S, ; IDEFICS Consortium Behavioural effects of a community-oriented setting–based intervention for prevention of childhood obesity in eight European countries: main results from the IDEFICS study. Obes Rev. 2015;16(S2)(suppl 2):30-40. doi:10.1111/obr.12347 26707014

[12] De Henauw S, Huybrechts I, De Bourdeaudhuij I, ; IDEFICS Consortium Effects of a community-oriented obesity prevention programme on indicators of body fatness in preschool and primary school children: main results from the IDEFICS study. Obes Rev. 2015;16(S2)(suppl 2):16-29. doi:10.1111/obr.12346 26707013

[13] Braun KL, Nigg CR, Fialkowski MK, Using the ANGELO model to develop the Children’s Healthy Living Program multilevel intervention to promote obesity preventing behaviors for young children in the US-Affiliated Pacific region. Child Obes. 2014;10(6):474-481. doi:10.1089/chi.2014.0102 25369548 PMC4267707

[14] Simmons A, Mavoa HM, Bell AC, Creating community action plans for obesity prevention using the ANGELO (Analysis Grid for Elements Linked to Obesity) Framework. Health Promot Int. 2009;24(4):311-324. doi:10.1093/heapro/dap029 19759046 PMC2776999

[15] Fialkowski MK, Yamanaka A, Wilkens LR, Recruitment strategies and lessons learned from the Children’s Healthy Living Program Prevalence Survey. AIMS Public Health. 2016;3(1):140-157. doi:10.3934/publichealth.2016.1.140 29546153 PMC5690270

[16] Fialkowski MK, DeBaryshe B, Bersamin A, ; CHL Team. A community engagement process identifies environmental priorities to prevent early childhood obesity: the Children’s Healthy Living (CHL) Program for remote underserved populations in the US Affiliated Pacific Islands, Hawaii and Alaska. Matern Child Health J. 2014;18(10):2261-2274. doi:10.1007/s10995-013-1353-3 24043557 PMC4220109

[17] Wilkens LR, Novotny R, Fialkowski MK, Children’s Healthy Living (CHL) Program for remote underserved minority populations in the Pacific region: rationale and design of a community randomized trial to prevent early childhood obesity. BMC Public Health. 2013;13(1):944. doi:10.1186/1471-2458-13-944 24107083 PMC3851862

[18] Colleges of agriculture at the land grant universities: public service and public policy: an excerpt from the Executive Summary of the National Research Council Report. Proc Natl Acad Sci U S A. 1997;94(5):1610-1611. doi:10.1073/pnas.94.5.1610 11607723 PMC34139

[19] Butel J, Braun KL, Novotny R, Assessing intervention fidelity in a multi-level, multi-component, multi-site program: the Children’s Healthy Living (CHL) Program. Transl Behav Med. 2015;5(4):460-469. doi:10.1007/s13142-015-0334-z 26622918 PMC4656219

[20] Glasgow RE, Vogt TM, Boles SM Evaluating the public health impact of health promotion interventions: the RE-AIM framework. Am J Public Health. 1999;89(9):1322-1327. doi:10.2105/AJPH.89.9.1322 10474547 PMC1508772

[21] Li F, Wilkens LR, Novotny R, Anthropometric measurement standardization in the US-Affiliated Pacific: report from the Children’s Healthy Living Program. Am J Hum Biol. 2016;28(3):364-371. doi:10.1002/ajhb.22796 26457888 PMC4861683

[22] Yamanaka A, Fialkowski MK, Wilkens L, et al. Quality assurance of data collection in the multi-site community randomized trial and prevalence survey of the Children’s Healthy Living Program. BMC Res Notes. 2016;9(1):432. doi:10.1186/s13104-016-2212-2 27590179 PMC5009559

[23] Centers for Disease Control and Prevention A SAS program for the 2000 CDC growth charts (ages 0 to <20 years). https://www.cdc.gov/nccdphp/dnpao/growthcharts/resources/sas.htm. Published 2018. Accessed April 6, 2018.

[24] Cook S, Auinger P, Huang TT Growth curves for cardio-metabolic risk factors in children and adolescents. J Pediatr. 2009;155(3):S6.e15-S6.e26. 10.1016/j.jpeds.2009.04.051 PMC2789447 19732566

[25] Zerfas AJ Checking Continuous Measures: Manual for Anthropometry. Los Angeles: Division of Epidemiology, School of Public Health, University of California; 1985.

[26] Burke JP, Hale DE, Hazuda HP, Stern MP A quantitative scale of acanthosis nigricans. Diabetes Care. 1999;22(10):1655-1659. doi:10.2337/diacare.22.10.1655 10526730

[27] McGreavey JA, Donnan PT, Pagliari HC, Sullivan FM The Tayside Children’s Sleep Questionnaire: a simple tool to evaluate sleep problems in young children. Child Care Health Dev. 2005;31(5):539-544. doi:10.1111/j.1365-2214.2005.00548.x 16101649

[28] Yonemori KM, Ennis T, Novotny R, Collecting wrappers, labels, and packages to enhance accuracy of food records among children 2-8 years in the Pacific region: Children’s Healthy Living Program (CHL). J Food Compost Anal. 2017;64(pt 1):112-118. doi:10.1016/j.jfca.2017.04.012 29398780 PMC5792074

[29] Novotny R, Nigg C, McGlone K, Pacific Tracker 2–Expert System (PacTrac2-ES) behavioural assessment and intervention tool for the Pacific Kids DASH for Health (PacDASH) study. Food Chem. 2013;140:471-477. doi:10.1016/j.foodchem.2012.11.047 23601394

[30] Martin CL, Murphy SP, Leon Guerrero RT, Davison N, Jung YO, Novotny R The Pacific Tracker (PacTrac): development of a dietary assessment instrument for the Pacific. J Food Compost Anal. 2008;21(suppl 2):S103-S108. doi:10.1016/j.jfca.2007.06.007 25729156 PMC4340481

[31] Dekkers AL, Verkaik-Kloosterman J, van Rossum CT, Ocké MC SPADE, a new statistical program to estimate habitual dietary intake from multiple food sources and dietary supplements. J Nutr. 2014;144(12):2083-2091. doi:10.3945/jn.114.191288 25320187

[32] Haas S, Nigg CR Construct validation of the stages of change with strenuous, moderate, and mild physical activity and sedentary behaviour among children. J Sci Med Sport. 2009;12(5):586-591. doi:10.1016/j.jsams.2008.11.001 19249241

[33] Ettienne R, Nigg CR, Li F, Validation of the Actical accelerometer in multiethnic preschoolers: the Children’s Healthy Living (CHL) Program. Hawaii J Med Public Health. 2016;75(4):95-100. 27099804 PMC4832876

[34] Baranowski T, Lytle L Should the IDEFICS outcomes have been expected? Obes Rev. 2015;16(suppl 2):162-172. doi:10.1111/obr.12359 26707025

[35] Veldhuis L, Vogel I, Renders CM, Behavioral risk factors for overweight in early childhood: the “Be active, eat right” study. Int J Behav Nutr Phys Act. 2012;9:74. doi:10.1186/1479-5868-9-74 22704042 PMC3409071

[36] Li P, Redden DT Comparing denominator degrees of freedom approximations for the generalized linear mixed model in analyzing binary outcome in small sample cluster-randomized trials. BMC Med Res Methodol. 2015;15:38. doi:10.1186/s12874-015-0026-x 25899170 PMC4458010

[37] McLaughlin LA, Braun KL Asian and Pacific Islander cultural values: considerations for health care decision making. Health Soc Work. 1998;23(2):116-126. doi:10.1093/hsw/23.2.116 9598394

