# Supplementary material for: Effect of the Children’s Healthy Living Program on Young Child Overweight, Obesity, and Acanthosis Nigricans in the US-Affiliated Pacific Region: A Randomized Clinical Trial
Source: JAMA Netw Open. 2018 Oct 26;1(6):e183896. doi: 10.1001/jamanetworkopen.2018.3896 (PMC6324447; doi:10.1001/jamanetworkopen.2018.3896)
Supplement: Supplement 2. — eAppendix. Supplemental Appendix eTable 1. Sample Size by Measurement Modules and by Intervention Time Period eTable 2. CHL Optimized Intervention of 8 Activities and 6 Behavioral Targets eTable 3. Children’s Healthy Living Program Trial Mean Changes in Primary and Secondary Outcomes Between Intervention and Temporal Communities, US-Affiliated Pacific Region, 2012-2015 eFigure. Children’s Healthy Living (CHL) Program (a) Acanthosis Nigricans and (b) Screen Time Comparisons Between Intervention and Control Groups With Age and Sex Interaction P Values, US-Affiliated Pacific, 2012-2015 [file jamanetwopen-1-e183896-s002.pdf]

Supplementary Online Content

Novotny R, Davis J, Butel J, et al. Effect of the Children's Healthy Living Program on young child overweight, obesity, and acanthosis nigricans in the US-Affiliated Pacific region: a randomized clinical trial. *JAMA Netw Open*. 2018;1(6):e183896. doi:10.1001/jamanetworkopen.2018.3896

eAppendix. Supplemental Appendix

eTable 1. Sample Size by Measurement Modules and by Intervention Time Period

eTable 2. CHL Optimized Intervention of 8 Activities and 6 Behavioral Targets

eTable 3. Children's Healthy Living Program Trial Mean Changes in Primary and Secondary Outcomes Between Intervention and Temporal Communities, US-Affiliated Pacific Region, 2012-2015

eFigure. Children's Healthy Living (CHL) Program (a) Acanthosis Nigricans and (b) Screen Time Comparisons Between Intervention and Control Groups With Age and Sex Interaction *P* Values, US-Affiliated Pacific, 2012-2015

This supplementary material has been provided by the authors to give readers additional information about their work.

© 2018 Novotny R et al. *JAMA Network Open*.

eAppendix. Supplemental Appendix

The hypothesis for the CHL group-randomized trial was communities that received the intervention would have less obesity in children than control communities or than background trends, as represented by Temporal communities. Therefore, the primary outcomes for the CHL intervention trial are body size variables. Secondary outcomes are the clinical manifestation in Acanthosis Nigricans and intermediate behaviors, including diet, physical activity and sleep. The hypothesis was tested using a hierarchical marginal model of the outcomes that accounted for the complex study design. See reference Wilken et al [1] for a full description of the study design. Briefly, five US Pacific jurisdictions were included in the intervention: the two states of Alaska and Hawaii, the two territories of Guam and American Samoa, and the Commonwealth of the Northern Marianas. Eligible communities were identified in each of the jurisdictions. Two pairs of communities in 4 jurisdictions and 1 pair of communities in Alaska were selected to be randomized to the Intervention or Control groups, after engagement with and agreement of the communities. The remaining eligible communities were used as Temporal communities to monitor trends in obesity over time. Fewer communities were included in Alaska due to the large distances to be covered.

Outcomes were regressed on the following community level variables: indicators for study group (Intervention, Control, Temporal), indicators for time (Baseline, Post-Intervention), and interaction terms between group and time. The intervention effect was tested within the strata of jurisdiction and accounting for the clustering of children within communities. Age and sex were adjusted for at the child level. Ethnicity/race was not adjusted for as it is highly confounded with jurisdiction. However, when the analysis was further adjusted for an indicator for the indigenous status of the child, the results were unaltered. The sample was also weighted to reflect the number of children residing in each community. The purpose of this weighting was to provide estimates of the outcomes that reflected the overall CHL region of underserved children. The number of children assessed across jurisdictions was similar with a ratio of the largest to smallest number of children assessed of 1.35. However, the ratio of the largest to smallest number of children residing in those jurisdictions was 17.4. Therefore, without weighting, the estimates of obesity, for instance, would heavily reflect the smallest jurisdiction of American Samoa rather than the largest of Alaska. Linear regressions were fit for continuous outcomes, and logistic regressions were for dichotomous outcomes. The expected means or proportions with 95% confidence intervals for each study group and each time point were estimated from the model. The test of the intervention was based on a “difference-in-difference” approach. Specifically, the Wald statistic of the interaction term for Intervention versus Control, or Intervention versus Temporal communities was used. As the unit of randomization was community, the degrees of freedom for this test was based on the number of communities (27 for most primary outcomes, 18 for most secondary

© 2018 Novotny R et al. *JAMA Network Open*.

outcomes). The model assumptions of normal and homoscedastic residuals were checked for the continuous outcomes. There were violations for four outcomes: waist circumference, screentime, Tayside sleep score, and water intake. Box-Cox transformations were applied to these outcome measures and the test of the hypothesis was performed on the transformed scale. For ease of interpretation, the expected means and 95% confidence intervals are presented in the natural units through back transformation. Specifically, if the outcome was transformed as f(Y), the model provides means M and confidence intervals M_{low} - M_{upper} on the transformed scale, we backtransformed the mean as $f^{-1}(M)$ and the confidence interval as $f^{-1}(M_{lower})$ to $f^{-1}(M_{upper})$. These analyses were performed using the SAS SURVEYREG procedure for continuous outcomes and SAS SURVEYLOGISTIC procedure for dichotomous outcomes, and statements for STRATA, CLUSTER and WEIGHT.

Reference

1. Wilken LR, Novotny R, Fialkowski MK, Boushey CJ, Nigg C, Paulino Y, Leon Guerrero R, Bersamin A, Vargo D, Kim J, Deenik J. Children's healthy living (CHL) program for remote underserved minority populations in the pacific region: rationale and design of a community randomized trial to prevent early childhood obesity. BMC Public Health. 2013;13(1):944. doi: 10.1186/1471-2458-13-944. PubMed PMID: 24107083

© 2018 Novotny R et al. *JAMA Network Open*.

Jurisdiction Information					
**Name**	**Alaska**	**American Samoa**	**Commonwealth of the Northern Mariana Islands**	**Guam**	**Hawaii**
Geographic coordinates	64.2008° N, 149.4937° W	14.2710° S, 170.1322° W	15.0979 N, 145.6739 E	13.4443° N, 144.7937° E	19.8968° N, 155.5828° W
Distance from coordinating center (Honolulu, HI)	3072 miles/North	2558 miles/Southwest	3864 miles/West	3950 miles/West	0.0 miles
US affiliation and land type description	A US state located in the North Pacific Ocean. Consists of the largest land area in the US	US territory located in the South Pacific Ocean. Consists of five islands and two coral atolls: Tutuila, with the Manu‘a Islands, Rose Atoll, and Swains Island.	US commonwealth territory of 15 volcanic islands in the Western Pacific Ocean. Consists of three major inhabited islands: Saipan, Tinian, and Rota.	US territory located in the Western Pacific Ocean. Consists of one island	A US state located in the Central Pacific Ocean. Consists of eight major islands: Hawaii, Maui, Molokai, Lanai, Kahoolawe, Oahu, Kauai, Niihau
Population size (2010 census)	713 985	55 519	53 883	159 358	1 360 301

© 2018 Novotny R et al. *JAMA Network Open*.

eTable 1. Sample Size by Measurement Modules and by Intervention Time Period

	Intervention		Control		Temporal		
**Measurement Modules**	Time 1	Time 2	Time 1	Time 2	Time 1	Time 2	**Total**
Demographic Questionnaire	1517	1343	1491	1295	1321	1405	8372
Context-Specific Questionnaires *	1516	1345	1491	1295	0	0	5647
Anthropometry	1512	1309	1486	1268	1319	1369	8263
Acanthosis Nigricans	1509	1320	1479	1278	0	0	5586
Food Activity Log	1343	1159	1195	1067	0	0	4764
Accelerometer	893	701	731	831	0	0	3156

*Acculturation; Screen-Time; Sleep Disturbance

© 2018 Novotny R et al. *JAMA Network Open*.

eTable 2. CHL Optimized Intervention of 8 Activities and 6 Behavioral Targets*

	↓ SSB	↑ F/V	↑ PA	↑ Water	↓ Screen	↑ Sleep
POLICY- Review Assessment Data & Identify areas for Improvement for Policy & Physical Environment related to 6 CHL behaviors						
a. Review preschool wellness policy assessment data to identify training needs	X	X	X	X	X	X
ENVIRONMENT- Partner and Advocate for Environmental Change						
a. Support environmental initiatives that relate to the CHL target behaviors	X	X	X	X	X	X
SOCIAL MARKETING- Promote the CHL Message						
a. Support role models to deliver CHL message	X	X	X	X	X	X
b. Promote CHL social marketing campaigns	X	X	X	X	X	X
CAPACITY BUILDING- Train the Trainers						
a. Train individuals to lead interactive, hands-on, & family-based sessions (to include gardening)	X	X	X	X	X	X
b. Train preschool providers on wellness policy implementation	X	X	X	X	X	X
c. Train role models (community champions, adult and youth role models)	X	X	X	X	X	X
d. Coalition development	X	X	X	X	X	X

*↓ - increase, ↑ - decrease, SSB - Sugar Sweetened Beverage Consumption; F/V - Fruit and Vegetable Consumption; PA - Physical Activity; Water - Water consumption; Screen - Leisure screen time; Sleep - Time spent sleeping

© 2018 Novotny R et al. *JAMA Network Open*.

eTable 3. Children's Healthy Living Program Trial Mean Changes in Primary and Secondary Outcomes Between Intervention and Temporal Communities, US-Affiliated Pacific Region, 2012-2015[a]

Child Measure	No.[b]	Temporal				Intervention vs Temporal	
		Time 1 (95% CI)	Time 2 (95% CI)	Diff (95% CI)	*P* Value	Diff (95% CI)	*P* Value
Anthropometry							
Overweight/Obese, %	7944	31.02 (24.41 to 38.51)	29.75 (21.83 to 39.12)	−1.27 (−28.82 to 26.28)	.84	−2.63 (−8.58 to 3.32)	.28
BMI z-score	7944	0.64 (0.45 to 0.83)	0.60 (0.37 to 0.83)	−0.04 (−0.13 to 0.06)	.42	−0.02 (−0.14 to 0.09)	.68
Waist, cm	7992	52.14 (50.89 to 53.48)	52.67 (51.03 to 54.49)	0.54 (−0.14 to 1.21)	.06	−0.65 (−1.79 to 0.49)	.13
Acanthosis nigricans, %	5585	-	-	-	-	-	-
Sleep							
Sleep, h/d[c]	7699	9.98 (9.69 to 10.27)	10.07 (9.54 to 10.59)	0.09 (−0.72 to 0.35)	.59	−0.02 (−0.53 to 0.49)	.93
Sleep disturbance score[1-9c]	5499	-	-	-	-	-	-
Screen, h/d[c]	5494	-	-	-	-	-	-
Accelerometer, min/d[d]							
Total METs	2951	-	-	-	-	-	-
Vigorous/Moderate	2951	-	-	-	-	-	-
Sedentary/Light[e]	2951	-	-	-	-	-	-
Diet, cups/d							
Fruit	4763	-	-	-	-	-	-
Vegetables	4763	-	-	-	-	-	-
Water[c]	4763	-	-	-	-	-	-
Sugar sweetened beverages	4763	-	-	-	-	-	-

Abbreviation: BMI, body mass index; METs, metabolic equivalent tasks.
[a]Means are based on a mixed model with a linear link for continuous outcomes and a logistic link for dichotomous outcomes that accounts for the randomization unit of community and the community clusters within jurisdiction strata, weights for the number of children in each community, and adjusts for child's age and sex. *P* values are based on a Wald test, with *df* based on the number of communities.

© 2018 Novotny R et al. *JAMA Network Open*.

[b]Number of children included in analysis.
[c]The variables were back transformed from the regression model.
[d]Number of minutes per day within bouts of 5 minutes, averaged over 4 to 6 days of accelerometer use.
[e]Sedentary and light includes sleep activity.

© 2018 Novotny R et al. *JAMA Network Open*.

eFigure. Children's Healthy Living (CHL) Program (a) Acanthosis Nigricans and (b) Screen Time Comparisons Between Intervention and Control Groups With Age and Sex Interaction *P* Values, US-Affiliated Pacific, 2012-2015

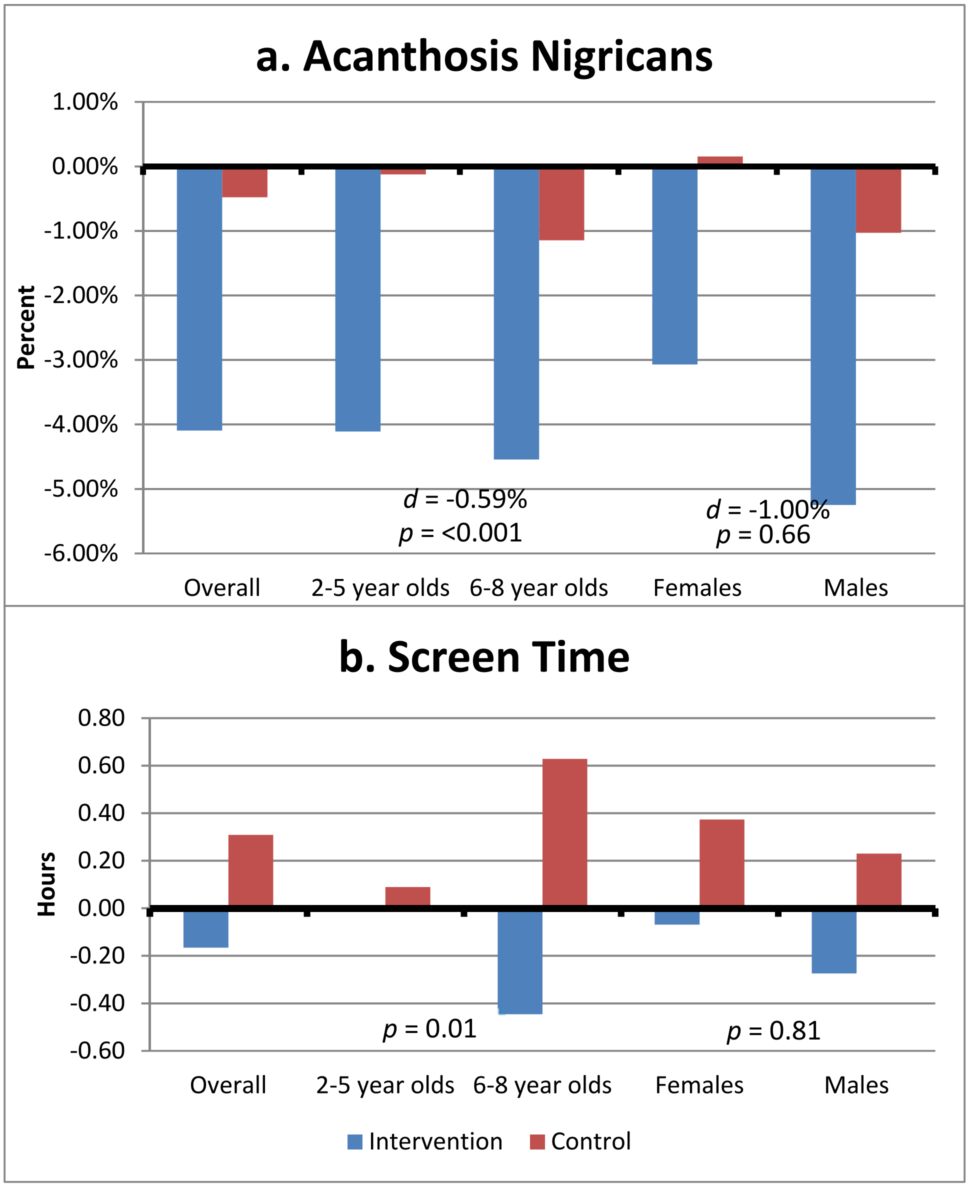

© 2018 Novotny R et al. *JAMA Network Open*.
