# Supplementary material for: Effect of the Children’s Healthy Living Program on Young Child Overweight, Obesity, and Acanthosis Nigricans in the US-Affiliated Pacific Region: A Randomized Clinical Trial
Source: JAMA Netw Open. 2018 Oct 26;1(6):e183896. doi: 10.1001/jamanetworkopen.2018.3896 (PMC6324447; doi:10.1001/jamanetworkopen.2018.3896)
Supplement: Supplement 3. — Data Sharing Statement [file jamanetwopen-1-e183896-s003.pdf]

Data Sharing Statement

Novotny. Effect of the Children’s Healthy Living Program on Young Child Overweight, Obesity, and Acanthosis Nigricans in the US-Affiliated Pacific Region. *JAMA Network Open*. Published October 26, 2018. 10.1001/jamanetworkopen.2018.3896

Data
Data available: Yes
Data types: Other (please specify)
Additional Information: Upon approval by Children's Healthy Living (CHL) Program Steering Committee, and with proof of adequate system for data security, data sets for specific research questions not already under investigation will be made available.
How to access data: See chl-pacific.org at the Data Request button.
When available: With publication

Supporting Documents
Document types: None

Additional Information
Who can access the data: Upon approval by Children's Healthy Living (CHL) Program Steering Committee, and with proof of adequate system for data security, data sets for specific research questions not already under investigation will be made available.
Types of analyses: Upon approval by Children's Healthy Living (CHL) Program Steering Committee, and with proof of adequate system for data security, data sets for specific research questions not already under investigation will be made available.
Mechanisms of data availability: Upon approval by Children's Healthy Living (CHL) Program Steering Committee, and with proof of adequate system for data security, data sets for specific research questions not already under investigation will be made available.
Any additional restrictions: Upon approval by Children's Healthy Living (CHL) Program Steering Committee, and with proof of adequate system for data security, data sets for specific research questions not already under investigation will be made available.
